# TOPPER: Topology Prediction of Transmembrane Protein Based on Evidential Reasoning

**DOI:** 10.1155/2013/123731

**Published:** 2013-01-17

**Authors:** Xinyang Deng, Qi Liu, Yong Hu, Yong Deng

**Affiliations:** ^1^School of Computer and Information Science, Southwest University, Chongqing 400715, China; ^2^School of Life Sciences and Biotechnology, Shanghai Jiao Tong University, Shanghai 200240, China; ^3^Department of Biomedical Informatics, Medical Center, Vanderbilt University, Nashville, TN 37235, USA; ^4^Institute of Business Intelligence and Knowledge Discovery, Guangdong University of Foreign Studies, Sun Yat-sen University, Guangzhou 510006, China; ^5^School of Engineering, Vanderbilt University, Nashville, TN 37235, USA

## Abstract

The topology prediction of transmembrane protein is a hot research field in bioinformatics and molecular biology. It is a typical pattern recognition problem. Various prediction algorithms are developed to predict the transmembrane protein topology since the experimental techniques have been restricted by many stringent conditions. Usually, these individual prediction algorithms depend on various principles such as the hydrophobicity or charges of residues. In this paper, an evidential topology prediction method for transmembrane protein is proposed based on evidential reasoning, which is called TOPPER (topology prediction of transmembrane protein based on evidential reasoning). In the proposed method, the prediction results of multiple individual prediction algorithms can be transformed into BPAs (basic probability assignments) according to the confusion matrix. Then, the final prediction result can be obtained by the combination of each individual prediction base on Dempster's rule of combination. The experimental results show that the proposed method is superior to the individual prediction algorithms, which illustrates the effectiveness of the proposed method.

## 1. Introduction

According to the present genome data, roughly 20–30% of the genes in a typical organism code for *α*-helical transmembrane (TM) protein [1–3]. Transmembrane protein is the principal executives of the biomembrane's functions and plays many important roles in cell such as substance transportation, and energy conversion. In order to explore the structure, function, and transmembrane mechanism of transmembrane protein, the topology prediction of transmembrane protein has been a hot field in bioinformatics and molecular biology [1, 2, 4].

**Figure 1:**
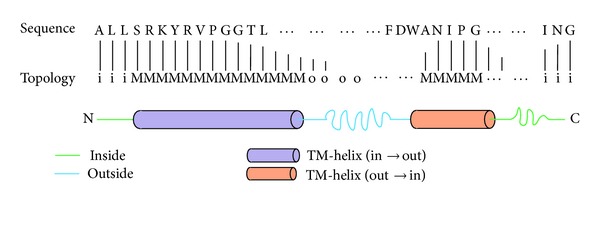
Topology prediction of transmembrane protein.

The topology of transmembrane protein [5], that is, the number and position of the transmembrane helixes and the in/out location of the N and C terminal of the protein sequence, is an important issue for the research of transmembrane proteins. For a protein sequence, if both transmembrane helixes and location of the N and C terminal have been predicted correctly, the topology of the protein sequence is said to be predicted correctly. Recently, information science and technology are widely used in the biology and medicine [6–8]. In essence, the topology prediction of transmembrane protein is a typical pattern recognition problem. As shown in Figure 1, given a protein sequence, the task is to determine the class label for each residue among these three classes of “i” (intracellular), “M” (transmembrane), and “o” (extracellular). At present, the most accurate methods to determine the topology of transmembrane protein are some experimental techniques, such as nuclear magnetic resonance (NMR) and X-ray crystal diffraction. However, these experimental techniques usually require strict conditions so that they cannot be applied on a large scale. They cannot meet the needs of the increasing protein sequences. Therefore, various computational methods have been developed to predict the topology of transmembrane protein [9–11].

Generally speaking, in a previous study there mainly exist three primary kinds of algorithms to predict the topology of transmembrane protein. The first kind of algorithms is on the basis of the chemical or physical properties of amino acids, for example, the hydrophobicity of residues or the charges of residues in different location. Some classical prediction algorithms are TopPred [2], and so on [12, 13]. The second kind of algorithms for the topology prediction is based on the statistical analysis on a huge amount of structure known as transmembrane proteins, such as MEMSAT [14], TMAP [10], and PRED-TMR [15]. In the third kind of algorithms, various machine learning technologies such as hidden Markov model (HMM) and support vector machine (SVM) have been introduced to the prediction of transmembrane protein topology. A series of algorithms have been developed, for example, HMMTOP [11], PHDhtm [16, 17], and so forth [18–21].

According to the mentioned above, even though there exists many algorithms for the prediction of transmembrane protein topology, however, different algorithms depend on different principles, and their applicable scopes are different. To a prediction system, if more information have been taken into consideration, the prediction ability of the system must be much more stronger. Essentially, it is a viewpoint of ensemble learning [22–25]. Using this idea to the topology prediction of transmembrane protein, various prediction algorithms have been treat as basic predictors; the task is the combination of multiple predictors to obtain a combination predictor which has a better performance than basic predictors. Within this process, there are two critical problems, that is, the representation of each predictor's prediction results and the combination method of combining multiple predictors. In regard to the representation of predictor's prediction results, as Xu et al. [23] pointed three types of output information can be utilized for different prediction algorithms, namely, the information in the abstract level, rank level, and measurement level, respectively. As to the combination method, traditional methodologies are usually on the basis of the framework of probability theory. To some degree, it is very effective, especially for the randomness. However, in the real world there are various uncertainties, not only the randomness but also the fuzziness and incompleteness, and so forth [26, 27].

As a theory of evidential reasoning under the uncertain environment, the Dempster-Shafer theory of evidence [28, 29] has an advantage of directly expressing various uncertainties and has been widely used in many fields [30–37]. It provides a general and effective framework for the representation and combination of multiple individual algorithms. In this paper, a new topology prediction method of transmembrane protein based on evidential reasoning approach, called TOPPER, has been proposed. In the proposed TOPPER method, the prediction results of basic predictor are represented by basic probability assignment (BPA) which has been constructed in terms of the confusion matrix of the predictor. Then, various basic predictors are combined by using the Dempster's rule of combination. Finally, the topology of a transmembrane protein sequence are determined according to the combination prediction results. In this paper, an experiment demonstrates the effectiveness of the propose prediction method.

The rest of this paper is organized as follows.  Section 2 introduces some basic concepts about the Dempster-Shafer theory of evidence. In Section 3 the proposed method is presented.  Section 4 gives experimental verification to demonstrate the effectiveness of the proposed method. Conclusions are given in Section 5.

## 2. Preliminaries

In this section, a few concepts commonly in the Dempster-Shafer theory of evidence will be introduced.

The Dempster-Shafer theory of evidence [28, 29], also called the Dempster-Shafer theory or evidence theory, is used to deal with uncertain information. As an effective theory of evidential reasoning, the Dempster-Shafer theory has an advantage of directly expressing various uncertainties. This theory needs weaker conditions than the Bayesian theory of probability, so it is often regarded as an extension of the bayesian theory. For completeness of the explanation, a few basic concepts are introduced as follows.

**Definition 1**

Let *Ω* be a set of mutually exclusive and collectively exhaustive, indicted by

(1) $$\begin{array}{c} \Omega =\left\{E_{1},E_{2},\ldots ,E_{i},\ldots ,E_{N}\right\}. \end{array}$$

The set *Ω* is called frame of discernment. The power set of *Ω* is indicated by 2^*Ω*^, where

(2) $$\begin{array}{c} 2^{\Omega }=\left\{\emptyset ,\left\{E_{1}\right\},\ldots ,\left\{E_{N}\right\},\left\{E_{1},E_{2}\right\},\ldots ,\left\{E_{1},E_{2},\ldots ,E_{i}\right\},\ldots ,\Omega \right\}. \end{array}$$

If *A* ∈ 2^*Ω*^, *A* is called a proposition.

**Definition 2**

For a frame of discernment *Ω*, a mass function is a mapping *m* from 2^*Ω*^ to [0,1], formally defined by

(3) $$\begin{array}{c} m:2^{\Omega }\to \left[0,1\right], \end{array}$$

which satisfies the following condition:

(4) $$\begin{array}{c} m\left(\emptyset \right)=0,\;\;\sum_{A\in 2^{\Omega }}m\left(A\right)=1. \end{array}$$

In the Dempster-Shafer theory, a mass function is also called a basic probability assignment (BPA). If *m*(*A*) > 0, *A* is called a focal element, the union of all focal elements is called the core of the mass function.

**Definition 3**

For a proposition *A*⊆*Ω*, the belief function Bel : 2^*Ω*^ → [0,1] is defined as

(5) $$\begin{array}{c} \text{Bel}\left(A\right)=\sum_{B\subseteq A}m\left(B\right). \end{array}$$

The plausibility function Pl : 2^*Ω*^ → [0,1] is defined as

(6) $$\begin{array}{c} \text{Pl}\left(A\right)=1-\text{Bel}\left(\overset{-}{A}\right)=\sum_{B\cap A\neq \emptyset }m\left(B\right), \end{array}$$

where $\overset{-}{A}=\Omega -A$.

Obviously, Pl(*A*) ≥ Bel(*A*); these functions Bel and Pl are the lower limit function and upper limit function of proposition *A*, respectively.

Consider two pieces of evidence indicated by two BPAs *m*_1_ and *m*_2_ on the frame of discernment *Ω*; the Dempster's rule of combination is used to combine them. This rule assumes that these BPAs are independent.

**Definition 4**

The Dempster's rule of combination, also called orthogonal sum, denoted by *m* = *m*_1_⨁*m*_2_, is defined as follows:

(7) $$\begin{array}{c} m\left(A\right)=\{\begin{array}{cc} \frac{1}{1-K}\sum_{B\cap C=A}m_{1}\left(B\right)m_{2}\left(C\right), & A\neq \emptyset ;\\ 0, & A=\emptyset , \end{array} \end{array}$$

with

(8) $$\begin{array}{c} K=\sum_{B\cap C=\emptyset }m_{1}\left(B\right)m_{2}\left(C\right). \end{array}$$

Note that the Dempster's rule of combination is only applicable to such two BPAs which satisfy the condition *K* < 1.

## 3. Proposed Method

In this section, a new transmembrane protein topology prediction method is proposed based on evidential reasoning. For the sake of convenience, it is briefly written down as TOPPER (Topology prediction of transmembrane protein based on evidential reasoning). The proposed prediction method TOPPER is on the basis of the combination of multiple individual prediction algorithms. In order to obtain the combination predictor, the process is presented step by step as follows.

### 3.1. The Selection of Basic Predictor

Because the proposed topology prediction method is the combination of multiple individual prediction methods, the basic predictors should be constructed first. Here, five individual prediction algorithms, OCTOPUS [3], PRO-TMHMM and PRODIV-TMHMM [38], SCAMPI-msa, and SCAMPI-seq [13], have been selected to construct these basic predictors. In pattern recognition, the prediction performance of each predictor is expressed by confusion matrix. In the topology prediction of transmembrane protein, since there are only three classes “i” (intracellular), “M” (transmembrane), and “o” (extracellular), the confusion matrix is formulated by

(9) $$\begin{array}{c} C_{\varphi }=\left[\begin{array}{ccc} n_{\text{ii}} & n_{\text{iM}} & n_{\text{io}}\\ n_{\text{Mi}} & n_{\text{MM}} & n_{\text{Mo}}\\ n_{\text{oi}} & n_{\text{oM}} & n_{\text{oo}} \end{array}\right], \end{array}$$

where each item *n*_*pq*_ is the number of residues belonging to the class *p* but predicted as the class *q* according to the basic predictor *φ*.

### 3.2. The Representation of the Basic Predictor's Prediction Results

In the combination of multiple predictors, the representation of the basic predictor's prediction results is a critical problem. In this paper, BPA is used to represent these prediction results. But the next is how to construct BPAs. For example, a residue in a protein sequence has been predicted that it belongs to transmembrane helix (i.e., class “M”) by a basic predictor. However, due to that the prediction is not 100% correct, how can we represent this uncertainty. Here, a classical and effective method proposed by Xu et al. [23] has been adopted to construct BPAs. In Xu et al.'s method, the output was treated as single class labels, and the source of evidence for the propositions of interest was defined on the basis of the performance of predictors in terms of recognition, substitution, and rejection rates which are generated from confusion matrix. Briefly speaking, it is a BPA construction method based on confusion matrix.

To a predictor of transmembrane protein topology *φ* with confusion matrix *C*_*φ*_, according to Xu et al.'s method [23], a BPA can be constructed for each class *p* by

(10) $$\begin{array}{c} m_{p}^{\varphi }\left(\left\{p\right\}\right)=R_{c}^{\varphi },\;\forall p\in \Omega ,\\ m_{p}^{\varphi }\left(\bar{\left\{p\right\}}\right)=1-R_{c}^{\varphi },\;\forall p\in \Omega ,\;\;\bar{\left\{p\right\}}=\frac{\Omega }{\left\{p\right\}}, \end{array}$$

with

(11) $$\begin{array}{c} R_{c}^{\varphi }=\frac{\sum_{p\in \Omega ,p=q}^{}n_{pq}}{\sum_{p\in \Omega }\sum_{q\in \Omega }^{}n_{pq}}, \end{array}$$

where *Ω* = {i, M, o}.

For a residue in a protein sequence, the constructed BPA is *m*_i_^*φ*^ if the prediction result shows that the residue belongs to class i. In two other situations of M and o, the constructed BPAs are *m*_M_^*φ*^ and *m*_o_^*φ*^, respectively.

### 3.3. The Combination of Multiple Predictors

**Figure 2:**
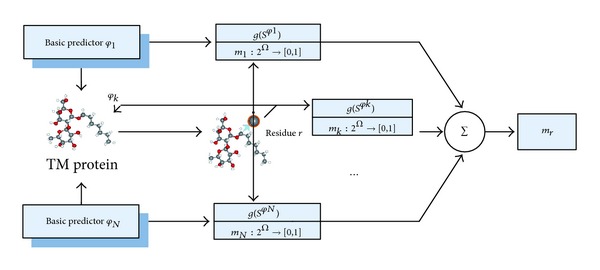
The combination of multiple predictors.

Once all BPAs of each predictor have been constructed, the prediction results of multiple predictors can be combined. In this paper, these prediction results of basic predictors have been treated as various evidences coming from different sources. The various prediction results can be combined by using the Dempster's rule of combination, as shown in Figure 2.

Assume there are *N* basic predictors in the evidential prediction system, *S* ^*φ*^ is the set of constructed BPAs for all classes from basic predictor *φ*, and *S* ^*φ*^ = {*m*_i_^*φ*^, *m*_M_^*φ*^, *m*_o_^*φ*^}. *g*(*S* ^*φ*^) is an operation used to obtain the matched BPA for a residue predicted by *φ*. The combination of multiple predictors to predict the class of residue *r* can be expressed by

(12) $$\begin{array}{c} m_{r}=g\left(S^{\varphi_{1}}\right)⨁g\left(S^{\varphi_{2}}\right)⨁\cdots ⨁g\left(S^{\varphi_{N}}\right). \end{array}$$

### 3.4. The Determination of Topology

Through the above steps, the combination prediction result has been derived for each residue in a transmembrane protein sequence. It is indicated by a BPA *m*_*r*_. In order to get the final class that the residue belongs to, the BPA will be translated into a probability distribution by using the so-called pignistic probability transformation (PPT) function, proposed by Smets and Kennes in the transferable belief model (TBM) [39]. The PPT function [39] is defined as follow.

Let *m* be a BPA on a frame of discernment *Ω*, a pignistic probability transformation function Bet*P*_*m*_ : *Ω* → [0,1] corresponding to *m* is

(13) $$\begin{array}{c} \text{Bet}P_{m}\left(x\right)=\sum_{A\subseteq \Omega ,x\in A}\frac{1}{\left|A\right|}\frac{m\left(A\right)}{1-m\left(\emptyset \right)},\;m\left(\emptyset \right)\neq 1, \end{array}$$

where |*A*| is the cardinality of proposition *A*.

By using PPT function, the BPA *m*_*r*_ can be translated into a probability distribution *p*_*r*_. Then the class of the residue *r* can be determined according to the maximum value of the probability distribution *p*_*r*_. At last, the topology of a transmembrane protein can be determined when the classes of all residues in the protein sequence have been determined. For each protein, the transmembrane orientation is determined by the location of the first residue, and each transmembrane region whose length exceeds a threshold consists of these residues labelled as class “M.” According to the topology, all transmembrane helixes and the orientation of each transmembrane helix can be derived.

## 4. Experimental Verification

In this paper, a data set of 125 transmembrane protein sequences with known topology is collected from the data set of MPtopo [40] to verify the effectiveness of the proposed method TOPPER.

In order to reflect the performance of combination predictor faithfully and to avoid overfitting, the experiment is performed using tenfold cross-validation. For each fold, it roughly contains 12-13 transmembrane proteins and their homology has been reduced to 30% below by using cd-hit program [41]. 

In order to assess the prediction performance of transmembrane regions (i.e., transmembrane helixes without considering orientation) of different algorithms, an evaluation method developed by Tusnády and Simon [11] is adopted in this paper. To a transmembrane region, the prediction is considered successful when the overlapping region of predicted and observed transmembrane region contains at least 9 amino acids. The total numbers of predicted and real observed transmembrane regions are indicated by *N*_prd_ and *N*_obs_, respectively. The overlapping predicted and real observed transmembrane regions are indicated by *N*_cor_. The efficiency of the transmembrane regions prediction is measured by *M* = *N*_cor_/*N*_obs_ and *C* = *N*_cor_/*N*_prd_. The overall prediction power is defined by

(14) $$\begin{array}{c} Q=\sqrt{M\cdot C}\times 100\%. \end{array}$$

Besides, if all transmembrane regions and orientation of a transmembrane protein sequence have been predicted correctly, the topology of the transmembrane protein is said to be predicted correctly.

In the rest of this section, various prediction algorithms will be compared from three aspects, namely, the prediction performance of residue level, transmembrane region level, and topology level, respectively.

**Figure 3:**
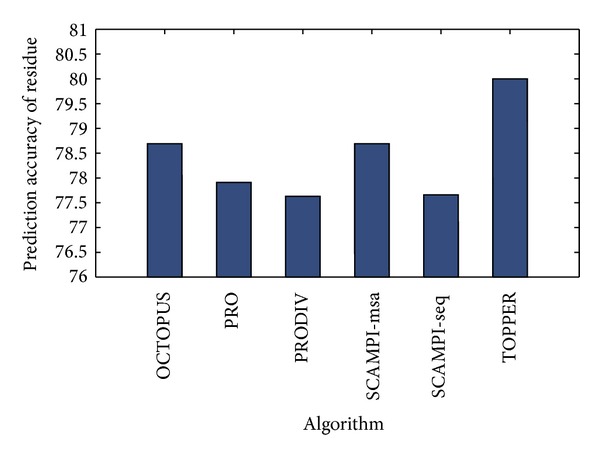
The comparison of residue's prediction accuracy between the proposed method and other algorithms.

**Figure 4:**
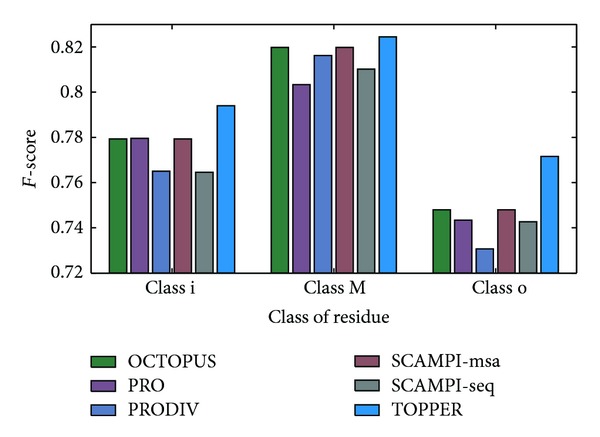
The comparison of *F* score between the proposed method and other algorithms.

In the level of residue prediction, the confusion matrix of residue prediction for each algorithm is shown in Table 1. According to these confusion matrices, Table 2 shows some indexes to measure the performance of residue prediction, including the recall rate, precision rate, *F* score of each class, and the prediction accuracy of residues. In TOPPER, the prediction accuracy of residue is 80.00%, while in other algorithms they are 78.69%, 77.91%, 77.63%, 78.69%, and 77.66%, respectively. The proposed method has the highest prediction accuracy of residue, shown in Figure 3. In addition, investigate the *F* score of each class in these algorithms. The TOPPER also has the highest value of *F* score no matter to class “i”, “M”, and “o”, shown in Figure 4. Hence, it is quite clear that the proposed TOPPER outperforms other algorithms.

**Figure 5:**
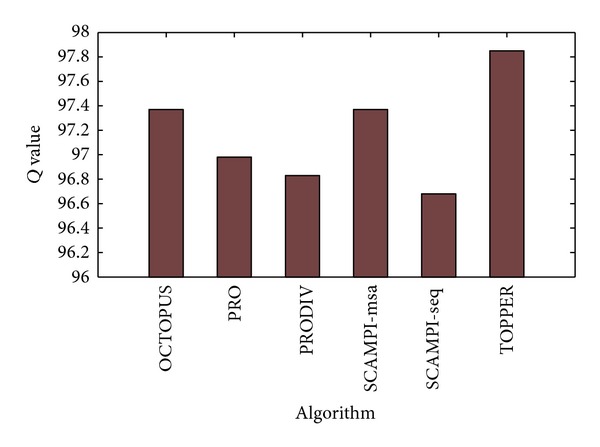
The comparison of transmembrane region's prediction performance between the proposed method and other algorithms.

In the level of transmembrane region prediction, Table 3 shows the prediction performance of various algorithms to the prediction of transmembrane region. According to the overall prediction power defined in [11], the *Q* value of TOPPER is 97.85%, while the *Q* values of other algorithms are 97.37%, 96.98%, 96.83%, 97.37%, and 96.68%, respectively. The *Q* value of TOPPER is the highest, shown in Figure 5. So TOPPER is superior to other algorithms.

**Figure 6:**
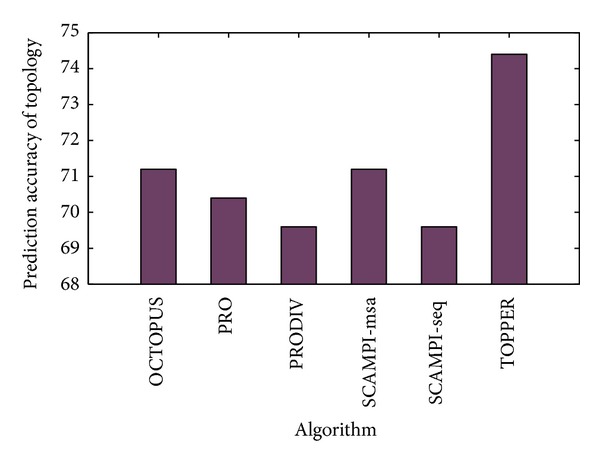
The comparison of topology's prediction accuracy between the proposed method and other algorithms.

In the level of topology prediction, Table 4 shows the prediction accuracy of topology for each algorithm. The topology's prediction accuracy of TOPPER is 74.4%, which is the highest among these algorithms, shown in Figure 6. Therefore, the proposed TOPPER is superior to other algorithms.

According to the mentioned above, the proposed TOPPER outperforms other algorithms no matter in the level of residue prediction, transmembrane region prediction, and topology prediction. Hence, the effectiveness of the proposed method has been demonstrated.

## 5. Conclusions

Transmembrane proteins are some special and important proteins in cells. The topology prediction of transmembrane protein is a foundation of the research of transmembrane proteins. In this paper, a new topology prediction method of transmembrane protein is proposed based on evidential reasoning. The proposed method is the combination of multiple individual prediction algorithms. In the proposed method, the Dempster-Shafer theory has been used to represent and combine the results of basic predictors. Experimental results show that the proposed method is superior to the individual prediction algorithms and demonstrates the effectiveness of the proposed method.

## Figures and Tables

**Table 1:** Confusion matrices of residue prediction for various algorithms.

Truth	Algorithm		Prediction	
		i	M	o
	OCTOPUS	7655	389	839
	PRO	7574	450	859
i	PRODIV	7323	442	1118
	SCAMPI-msa	7655	389	839
	SCAMPI-seq	7359	455	1069
	TOPPER	7636	358	889

	OCTOPUS	1877	9785	1458
	PRO	1922	9588	1610
M	PRODIV	1819	9884	1417
	SCAMPI-msa	1877	9785	1458
	SCAMPI-seq	1907	9628	1585
	TOPPER	1799	9817	1504

	OCTOPUS	1230	578	6091
	PRO	1051	714	6134
o	PRODIV	1117	775	6007
	SCAMPI-msa	1230	578	6091
	SCAMPI-seq	1101	564	6234
	TOPPER	916	518	6465

**Table 2:** Prediction performance of various algorithms in residue level.

Algorithm	Class	Recall (%)	Precision (%)	*F* score	Accuracy (%)
	i	86.18	71.13	0.7793	
OCTOPUS	M	74.58	91.01	0.8198	78.69
	o	77.11	72.62	0.7480	

	i	85.26	71.81	0.7796	
PRO	M	73.08	89.17	0.8033	77.91
	o	77.66	71.30	0.7434	

	i	82.44	71.38	0.7651	
PRODIV	M	75.34	89.04	0.8162	77.63
	o	76.05	70.32	0.7307	

	i	86.18	71.13	0.7793	
SCAMPI-msa	M	74.58	91.01	0.8198	78.69
	o	77.11	72.62	0.7480	

	i	82.84	70.98	0.7646	
SCAMPI-seq	M	73.38	90.43	0.8102	77.66
	o	78.92	70.14	0.7427	

	i	85.96	73.77	0.7940	
TOPPER	M	74.82	91.81	0.8245	80.00
	o	81.85	72.98	0.7716	

**Table 3:** Prediction performance of various algorithms in transmembrane region level.

Algorithm	*N*_obs_	*N*_prd_	*N*_cor_	*M* (%)	*C* (%)	*Q* (%)
OCTOPUS	515	512	500	97.09	97.66	97.37
PRO	515	512	498	96.70	97.27	96.98
PRODIV	515	524	503	97.67	95.99	96.83
SCAMPI-msa	515	512	500	97.09	97.66	97.37
SCAMPI-seq	515	507	494	95.92	97.44	96.68
TOPPER	515	507	500	97.09	98.62	97.85

**Table 4:** Prediction performance of various algorithms in topology level.

Algorithm	Prediction accuracy of topology (%)
OCTOPUS	71.2
PRO	70.4
PRODIV	69.6
SCAMPI-msa	71.2
SCAMPI-seq	69.6
TOPPER	74.4

## References

[1] Krogh A, Larsson B, Von Heijne G, Sonnhammer ELL (2001). Predicting transmembrane protein topology with a hidden Markov model: application to complete genomes. *Journal of Molecular Biology*.

[2] Von Heijne G (1992). Membrane protein structure prediction. Hydrophobicity analysis and the positive-inside rule. *Journal of Molecular Biology*.

[3] Viklund H, Elofsson A (2008). OCTOPUS: improving topology prediction by two-track ANN-based preference scores and an extended topological grammar. *Bioinformatics*.

[4] Honig B (2002). Combining bioinformatics and biophysics to understand protein-protein and protein-ligand interactions. *The Scientific World Journal*.

[5] Von Heijne G (2006). Membrane-protein topology. *Nature Reviews Molecular Cell Biology*.

[6] Tian L-P, Liu L-Z, Zhang Q-W, Wu F-X (2011). Nonlinear model-based method for clustering periodically expressed genes. *The Scientific World Journal*.

[7] Lightfoot AJ, Rosevear HM, O’Donnell MA (2011). Recognition and treatment of BCG failure in bladder cancer. *The Scientific World Journal*.

[8] Ercole B, Parekh DJ (2011). Methods to predict and lower the risk of prostate cancer. *The Scientific World Journal*.

[9] Melén K, Krogh A, Von Heijne G (2003). Reliability measures for membrane protein topology prediction algorithms. *Journal of Molecular Biology*.

[10] Persson B, Argos P (1996). Topology prediction of membrane proteins. *Protein Science*.

[11] Tusnády GE, Simon I (1998). Principles governing amino acid composition of integral membrane proteins: application to topology prediction. *Journal of Molecular Biology*.

[12] Kyte J, Doolittle RF (1982). A simple method for displaying the hydropathic character of a protein. *Journal of Molecular Biology*.

[13] Bernsel A, Viklund H, Falk J, Lindahl E, Von Heijne G, Elofsson A (2008). Prediction of membrane-protein topology from first principles. *Proceedings of the National Academy of Sciences of the United States of America*.

[14] Jones DT, Taylor W, Thornton J (1994). A model recognition approach to the prediction of all-helical membrane protein structure and topology. *Biochemistry*.

[15] Pasquier C, Promponas VJ, Palaios GA, Hamodrakas JS, Hamodrakas SJ (1999). A novel method for predicting transmembrane segments in proteins based on a statistical analysis of the SwissProt database: the PRED-TMR algorithm. *Protein Engineering*.

[16] Rost B, Casadio R, Fariselli P, Sander C (1995). Transmembrane helices predicted at 95% accuracy. *Protein Science*.

[17] Rost B, Casadio R, Fariselli P (1996). Refining neural network predictions for helical transmembrane proteins by dynamic programming. *Proceedings of the International Conference on Intelligent Systems for Molecular Biology*.

[18] Liu Q, Zhu YS, Wang BH, Li YX (2003). A HMM-based method to predict the transmembrane regions of *β*-barrel membrane proteins. *Computational Biology and Chemistry*.

[19] Deng Y, Liu Q, Li YX (2004). Scoring hidden Markov models to discriminate *β*-barrel membrane proteins. *Computational Biology and Chemistry*.

[20] Nugent T, Jones DT (2009). Transmembrane protein topology prediction using support vector machines. *BMC Bioinformatics*.

[21] Wang J, Li Y, Wang Q (2012). Pro- ClusEnsem: predicting membrane protein types by fusing different modes of pseudo amino acid composition. *Computers in Biology and Medicine*.

[22] Kittler J, Hatef M, Duin RPW, Matas J (1998). On combining classifiers. *IEEE Transactions on Pattern Analysis and Machine Intelligence*.

[23] Xu L, Krzyzak A, Suen CY (1992). Methods of combining multiple classifiers and their applications to handwriting recognition. *IEEE Transactions on Systems, Man and Cybernetics*.

[24] Wong W, Fos PJ, Petry FE (2003). Combining the performance strengths of the logistic regression and neural network models: a medical outcomes approach. *The Scientific World Journal*.

[25] Kusonmano K, Netzer M, Baumgartner C, Dehmer M, Liedl KR, Graber A (2012). Effects of pooling samples on the performance of classification algorithms: a comparative study. *The Scientific World Journal*.

[26] Barbosa AM, Real R (2012). Applying fuzzy logic to comparative distri- bution modelling: a case study with two sympatric amphibians. *The Scientific World Journal*.

[27] Al-Mubaid H, Gungu S (2012). A learning-based approach for biomedical word sense disambiguation. *The Scientific World Journal*.

[28] Dempster AP (1967). Upper and lower probabilities induced by a multivalued mapping. *Annals of Mathematics and Statistics*.

[29] Shafer G (1976). *A Mathematical Theory of Evidence*.

[30] Deng Y, Sadiq R, Jiang W, Tesfamariam S (2011). Risk analysis in a linguistic environment: a fuzzy evidential reasoning-based approach. *Expert Systems with Applications*.

[31] Yong D, WenKang S, ZhenFu Z, Qi L (2004). Combining belief functions based on distance of evidence. *Decision Support Systems*.

[32] Deng Y, Chan FTS (2011). A new fuzzy dempster MCDM method and its application in supplier selection. *Expert Systems with Applications*.

[33] Deng Y, Chan FTS, Wu Y, Wang D (2011). A new linguistic MCDM method based on multiple-criterion data fusion. *Expert Systems with Applications*.

[34] Deng Y, Jiang W, Sadiq R (2011). Modeling contaminant intrusion in water distribution networks: a new similarity-based DST method. *Expert Systems with Applications*.

[35] Deng Y, Chen Y, Zhang Y, Mahadevan S (2012). Fuzzy Dijkstra algorithm for shortest path problem under uncertain environment. *Applied Soft Computing*.

[36] Zhang Y, Deng X, Wei D, Deng Y (2012). Assessment of E-Commerce security using AHP and evidential reasoning. *Expert Systems with Applications*.

[37] Kang B, Deng Y, Sadiq R, Mahadevan S (2012). Evidential cognitive maps. *Knowledge-Based Systems*.

[38] Viklund H, Elofsson A (2004). Best *α*-helical transmembrane protein topology predictions are achieved using hidden Markov models and evolutionary information. *Protein Science*.

[39] Smets P, Kennes R (1994). The transferable belief model. *Artificial Intelligence*.

[40] Jayasinghe S, Hristova K, White SH (2001). MPtopo: a database of membrane protein topology. *Protein Science*.

[41] Li W, Godzik A (2006). Cd-hit: a fast program for clustering and comparing large sets of protein or nucleotide sequences. *Bioinformatics*.

